# Investigation of the Internal Structure of Hard-to-Reach Objects Using a Hybrid Algorithm on the Example of Walls

**DOI:** 10.3390/e27050534

**Published:** 2025-05-16

**Authors:** Rafał Brociek, Józef Szczotka, Mariusz Pleszczyński, Francesca Nanni, Christian Napoli

**Affiliations:** 1 Department of Artificial Intelligence Modelling, Faculty of Applied Mathematics, Silesian University of Technology, 44-100 Gliwice, Poland; rafal.brociek@polsl.pl; 2 Building Investments, Miodowa 31, 43-426 Gumna, Poland; 3 Department of Mathematical Methods in Technology and Computer Science, Faculty of Applied Mathematics, Silesian University of Technology, 44-100 Gliwice, Poland; 4 Department of Enterprise Engineering ‘Mario Lucertini’, University of Rome ‘Tor Vergata’ and INSTM RU Roma-Tor Vergata, Via del Politecnico 1, 00133 Rome, Italy; francesca.nanni@aid.difesa.it; 5 Department of Computer, Control and Management Engineering, Sapienza University of Rome, Via Ariosto 25, 00185 Roma, Italy; cnapoli@diag.uniroma1.it; 6 Institute for Systems Analysis and Computer Science, Italian National Research Council, Via dei Taurini 19, 00185 Roma, Italy; 7 Department of Computational Intelligence, Czestochowa University of Technology, 42-201 Czestochowa, Poland

**Keywords:** computed tomography, inverse problem, optimization, incomplete data set

## Abstract

The article presents research on the application of computed tomography with an incomplete dataset to the problem of examining the internal structure of walls. The case of incomplete information in computed tomography often occurs in various applications, e.g., when examining large objects or when examining hard-to-reach objects. Algorithms dedicated to this type of problem can be used to detect anomalies (defects, cracks) in the walls, among other artifacts. Situations of this type may occur, for example, in old buildings, where special caution should be exercised. The approach presented in the article consists of a non-standard solution to the problem of reconstructing the internal structure of the tested object. The classical approach involves constructing an appropriate system of equations based on X-rays, the solution of which describes the structure. However, this approach has a drawback: solving such systems of equations is computationally very complex, because the algorithms used, combined with incomplete information, converge very slowly. In this article, we propose a different approach that eliminates this problem. To simulate the structure of the tested object, we use a hybrid algorithm that is a combination of a metaheuristic optimization algorithm (Group Teaching Optimization Algorithm) and a numerical optimization method (Hook-Jeeves method). In order to solve the considered inverse problem, a functional measuring the fit of the model to the measurement data is created. The hybrid algorithm presented in this paper was used to find the minimum of this functional. This paper also shows computational examples illustrating the effectiveness of the algorithms.

## 1. Introduction

Knowledge of the structure of internal walls is very important in protecting buildings against the effects of moisture on walls. The internal structure of walls is often very complicated, which makes it very difficult to create a proper horizontal insulating membrane that protects the wall against moisture rising from the ground by capillary action.

The oldest way to protect walls against ground moisture was to make the ground floor walls from water-resistant stone. The most common material used was granite. Most of the structures built on such foundations have survived to this day, although the walls above the ground were often rebuilt and the foundations have survived in good condition. Such walls are characterized by very good resistance to moisture.

As the demand for building materials increased, stone was replaced and supplemented with ceramic materials. In historic buildings, you can most often find mixed walls on the ground floor, which greatly complicates diagnostics related to the correct implementation of horizontal insulation.

Most often, ceramic bricks with lime mortar were used to build walls. In the description of the phenomenon of moisture and heat transport, geometric parameters such as the effective radius of capillaries, tortuosity, porosity, and specific surface play an important role. The phenomenon of dampness and saturation is closely related to the structure of bodies. The pore size distribution determines the possibility of liquid movement. The structure of a wall is porous. The ratio of pore volume to skeleton, i.e., the space occupied by a solid, varies greatly. It is also worth noting that in such porous materials, especially aged or heterogeneous masonry, moisture transport does not always follow classical Fickian diffusion. Recent studies have demonstrated that in materials with complex internal pore structures, such as chalk-like rocks, moisture and solute transport exhibit anomalous diffusion behavior, which deviates significantly from normal expectations and can be described more accurately using fractional diffusion models or Continuous Time Random Walk (CTRW) frameworks [1]. These insights are crucial when analyzing historic masonry, where similar structural irregularities, dead-end pores, and tortuous paths are common and significantly affect the accuracy of predictive models based on incomplete tomographic data.

**Figure 1:**
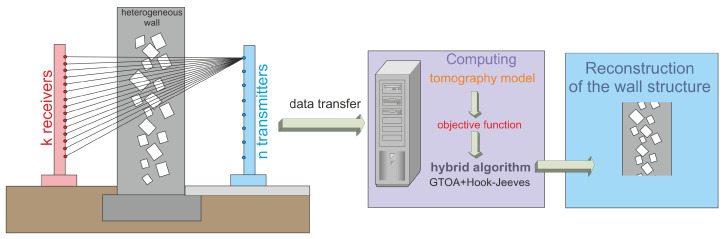
General scheme for determining the reconstruction of the structure of a non-uniform wall (containing voids, defects) based on the use of a hybrid algorithm (heuristic algorithm + deterministic algorithm).

In historic buildings, due to historical values, excavations cannot be made to check the construction of the wall or check the homogeneity of the wall. There are often voids in the walls as well as different wall compositions (so-called non-uniform walls). Therefore, it is important to perform a wall diagnosis using a non-invasive method. In this article, a tomography method with an incomplete set of information is proposed used to detect voids in walls. Figure 1 shows a diagram presenting the concept of operation of the proposed system for detecting voids in walls with non-uniform structure. The image showing the internal structure of the wall is determined based on the operation of a hybrid algorithm that is a combination of the heuristic algorithm and the Hook-Jeeves iterative algorithm.

In many technical and engineering issues, there is a need to study the internal structure of certain objects without disturbing their structure. The simplest example of this type of need is examining the inside of the human body. The classic non-invasive solution is an X-ray or computed tomography (CT) examination. Such a task has been developed for many years: the mathematical foundations were established at the beginning of the 20th century [2], long before the first computed tomography scanner was created. It was only in the mid-20th century, when the first computing machines were created, that these computationally complex algorithms could come into use (the first patient examination took place in 1971). The intensive development of both computed tomography algorithms and the rapid development of computer capabilities have long led to the fact that examinations of the human body at the resolution required by a doctor are carried out almost in real time. Therefore, contemporary work on computed tomography focuses mainly not on the reconstruction process itself, the development of algorithms, or increasing the speed of their convergence, but, among others, on image analysis (often using artificial intelligence) or the impact of reducing the radiation dose (which is important for patients’ health) on the quality of the obtained images [3,4,5].

Tomographic examination of a human being, however, fundamentally differs from tomographic examination of objects such as a heterogeneous wall. The mentioned mathematical foundations of computed tomography assume that to reconstruct the structure of the examined object, appropriate quality scans must be performed. Appropriate quality means not only small measurement errors but also a sufficient number of scanning angles and a sufficient number of rays for each of the mentioned angles. Such comfort is achievable when examining the human body, but when examining hard-to-reach or large objects, it is impossible to obtain scans of this quality. In such cases, we are dealing with the problem of incomplete datasets. For example, in the examination of heterogeneous walls, such scans can only be made from one side of the wall to the other (e.g., from the front of the wall to its back). Due to such limited data, the question arises: can such information guarantee the desired quality? The research conducted so far by the authors shows that the answer to this question is affirmative. However, the incomplete data set causes the algorithm reconstructing the structure of the examined object to converge slowly.

In this work, we attempt to eliminate the aforementioned problem. In the process of reconstructing the interior of a defensive wall, we use selected heuristic and determinist algorithms (discussed in detail in Section 4), thereby avoiding the computed-intensive part of the image reconstruction process and solving the corresponding system of linear equations (see Section 2).

This article proposes an innovative approach to the problem of reconstructing the internal structure of a wall based on an incomplete set of tomographic data. The main components of the work include: identifying the issue of incomplete measurement data, designing a functional that evaluates the fit of the model to real data, and constructing a hybrid optimization algorithm that combines the metaheuristic Group Teaching Optimization Algorithm (GTOA) with the deterministic Hooke-Jeeves method. The goal of the proposed algorithm is to efficiently minimize the aforementioned functional, enabling faster and more effective reconstruction of the analyzed object’s structure compared to classical methods. The effectiveness of the method is illustrated later in the article through computational examples. The key elements of the work are: identification of the data incompleteness problem, design of the fitting functional, construction of the hybrid algorithm, and demonstration of its effectiveness.

## 2. Computed Tomography

The applications of computed tomography (CT) are very broad, and generally speaking, it can be used wherever there is a need to examine the interior of an object without disrupting its structure. The primary field of application for CT methods is the examination of the human body, where the issue of incomplete datasets (as understood in this paper) does not arise. Less classical applications of CT include baggage inspections, geological and seismological studies, crash tests, and even space research [6,7,8].

Computed tomography operates on the principle that every type of matter has its own ability to absorb the energy of penetrating radiation passing through it. Based on this, by sending a stream of penetrating radiation with a known initial intensity and measuring the intensity of this stream after it has passed through the examined object, one can determine the type and amount of matter that was in the path of the radiation by calculating the amount of energy loss. Using the data collected in this manner and applying an appropriate algorithm, information about the examined object can be obtained. These algorithms can be divided into two groups: analytical algorithms and algebraic algorithms.

The first group of algorithms cannot handle the problem of an incomplete dataset (a classic example of such an algorithm is the backprojection algorithm [9]). Therefore, they can be successfully used, for example, in the examination of the human body, but we will not achieve good quality reconstruction when attempting to examine, for instance, a heterogeneous wall.

The second group of algorithms (algebraic algorithms, many of which are part of the ART family based on Kaczmarz’s method [10]) can handle incomplete information. The idea behind these algorithms is to reduce the problem of reconstructing the object’s structure to solving a system of linear equations. This is achieved by dividing the examined object into pixels, each assumed to be homogeneous (the pixel consists of only one type of matter). This approach allows the total energy loss of a ray to be decomposed into the sum of the energy losses occurring separately in each pixel through which the ray passes. Such a loss is proportional to the known (determinable) length of the ray’s path through a given pixel and the unknown (to be found) energy absorption value of that pixel (i.e., the type of matter composing the pixel). In this approach, the complexity of the task depends on the number of scans performed and the assumed resolution of the solution—the number of pixels determines this resolution. It is therefore easy to generate a system of equations (such a system has a specific form, which means that classical methods for solving systems of linear equations may struggle with it) that can have tens of thousands of unknowns and hundreds of thousands of rows. The more unilateral the scans are (as is the case, for example, in examining heterogeneous walls), the more slowly the algorithm for solving the resulting system of equations converges.

One attempt to avoid this issue could be the use of parallel computing (using appropriate algorithms designed for this purpose). The authors have studied such an approach, which proved to improve results (in terms of performance), but it still left much to do [11,12].

In this article, we propose a completely different approach to the problem of CT studies with an incomplete data set, which eliminates the issues present in the methods used so far.

## 3. Description of the Solution

As mentioned in the introduction, the complexity of the process of reconstructing the interior of the examined object in algebraic CT algorithms (which are the only ones capable of dealing with the problem of an incomplete data set) depends significantly on the accuracy (resolution) of the reconstructed image, i.e., on the predetermined number of pixels. An appropriate approach to this aspect of the problem can eliminate the previously encountered issues. Such an approach involves the use of heuristic algorithm.

One might think that this is not an innovative approach; many studies can be found where authors have used heuristic algorithms or machine learning in the field of computed tomography. However, a closer examination of these works will reveal that the mentioned algorithms are not used by the authors for image reconstruction, but rather for the analysis of an already reconstructed image, and the reconstruction process itself either occurs classically or is not of interest to the authors, as they assume that such an image already exists [13,14,15].

**Figure 2:**
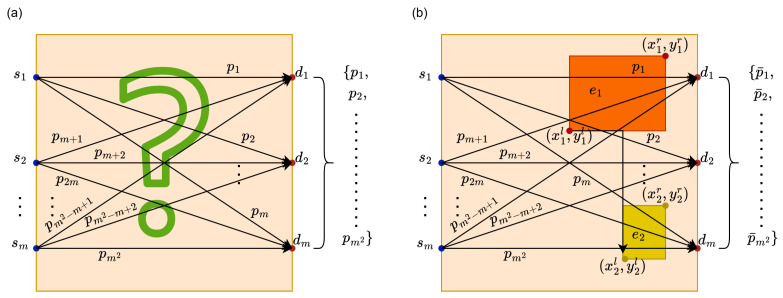
The diagram illustrates the formation of the actual projection vector (**a**) and the vector obtained from simulation (**b**).

In this paper, a heuristic algorithm was used at the stage of image creation. Of course, this cannot be achieved in a way that would involve solving linear equations using it; no heuristic algorithm can handle such a large number of dimensions. Our approach is based on the physical properties of the examined object. This object (we analyze the internal structure of heterogeneous walls) is mostly uniform, and in the examined fragment, only a few elements may appear (if they do) that differ from the background (by background, we mean the material constituting the uniform structure of the wall). We can assume that each of these objects can be represented by a certain rectangle $R_{i}$, i=1,2,…,n, which is uniquely determined by its position (four parameters corresponding to the coordinates of its vertices: bottom-left $x_{i}^{l}$, $y_{i}^{l}$, top-right $x_{i}^{r}$, $y_{i}^{r}$) and its composition (the fifth parameter corresponding to the type of matter from which the object is built, and thus its individual energy absorption capacity $e_{i}\geq 0$). Such an assumption allows for effective use of heuristic algorithms because the problem thus formulated will have 5n dimensions, where *n* in real cases should not exceed the value of 3. These algorithms will search for the appropriate vector in the space $R^{5n}$, describing the positions of the sought rectangles and the type of matter they are composed of. The assessment of the fit of the found vector will involve comparing it (in a certain sense) with a “reference vector”. Of course, we do not know this vector directly, but we know what projections are generated by it (by projection, we mean the energy loss of a single vector). We obtain such projections by scanning the examined object. The projection vector generated by the vector found through heuristic algorithms will be obtained through simulation—knowing the positions of radiation sources and detectors, the (external) shape of the examined object, and the position and composition of the generated objects $R_{i}$, we can conduct a simulation of scans and thus obtain the required projection vector. This situation is depicted in Figure 2.

In Figure 2, $s_{i}$, i=1,2,…,m denote consecutive radiation sources, $p_{i}$, $i=1,2,\ldots ,m^{2}$ represent consecutive projections (values of energy loss for the *i*-th ray) obtained from the real (examined) object, ${\bar{p}}_{i}$, $i=1,2,\ldots ,m^{2}$ denote consecutive projections (values of energy loss for the *i*-th ray) obtained from the simulated (generated by the appropriate heuristic algorithm) object, $d_{i}$, i=1,2,…,m are consecutive radiation detectors, $\left(x_{i}^{l},y_{i}^{l}\right)$, i=1,2 are the bottom-left coordinates of the rectangle $R_{i}$ generated by the heuristic algorithm, and $\left(x_{i}^{r},y_{i}^{r}\right)$ are the top-right vertices, while $e_{i}$ and i=1,2, correspond to the type of matter from which the object $R_{i}$ is built.

Having obtained the vectors $P=[p_{1},p_{2},\ldots ,p_{m^{2}}]$ and $\bar{P}=\left[{\bar{p}}_{1},{\bar{p}}_{2},\ldots ,{\bar{p}}_{m^{2}}\right]$, we can construct the functional F:

(1) $$F=∥P-\bar{P}∥=\sqrt{\sum_{i=1}^{m^{2}}{\left(p_{i}-{\bar{p}}_{i}\right)}^{2}},$$

which must be minimized to solve the problem.

**Figure 3:**
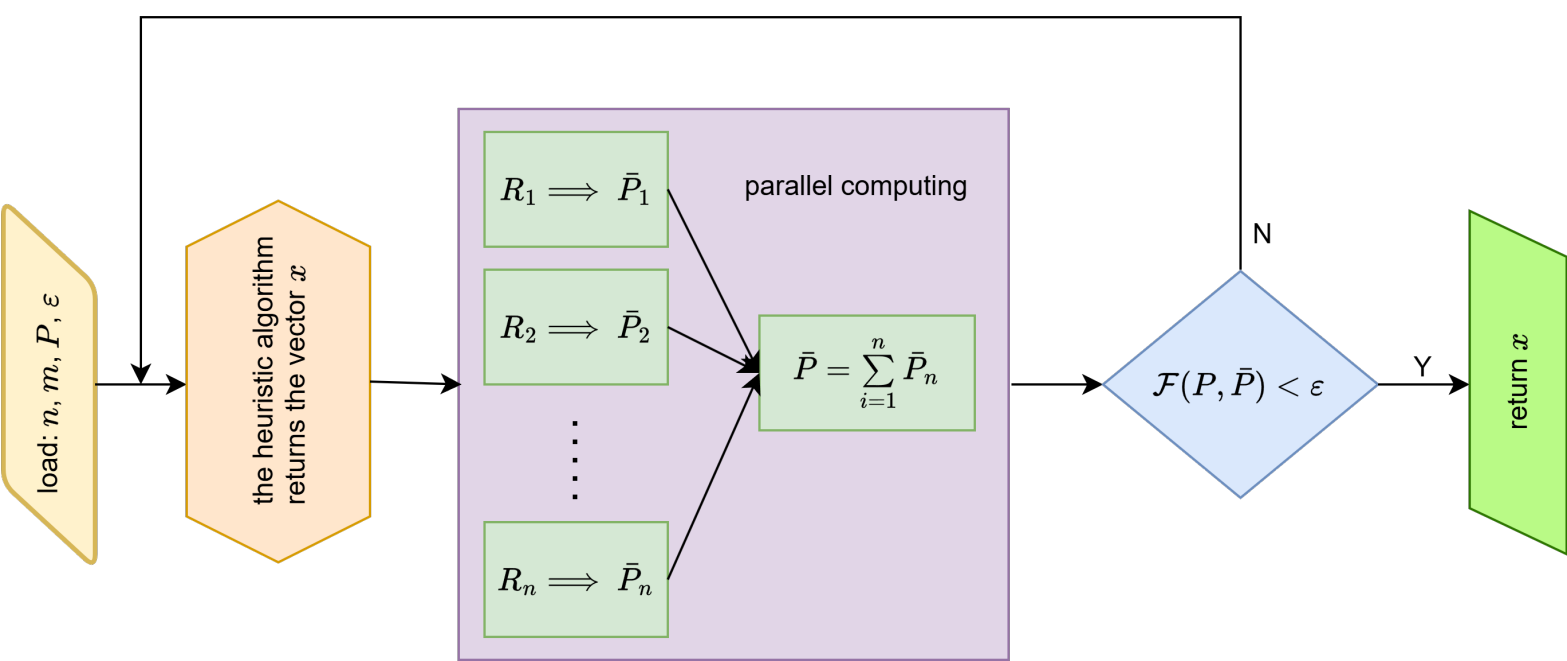
Block diagram of the process of reconstructing the interior of the tested object.

In general, the reconstruction process is depicted in Figure 3. After obtaining the projection vector *P* from real scans, the heuristic algorithm generates the vector *x* describing the structure of the examined object

$$x=[x_{1}^{l},y_{1}^{l},x_{1}^{r},y_{1}^{r},e_{1},x_{2}^{l},y_{2}^{l},x_{2}^{r},y_{2}^{r},e_{2},\ldots ,x_{n}^{l},y_{n}^{l},x_{n}^{r},y_{n}^{r},e_{n}].$$

From this vector, appropriate elements corresponding to the consecutive objects $R_{i}$, i=1,2,…,n are extracted. Then, for each of them, a projection vector is generated (via simulation), and since these operations are independent for each object, these calculations can be performed in parallel. The resulting partial vectors ${\bar{P}}_{i}$, i=1,2,…,n, are then summed, yielding an approximate solution $\bar{P}$. If this solution satisfies the condition $F(P,\bar{P})<\varepsilon$, where ε is a given accuracy, we accept this vector (and thus this distribution of matter in the examined object) as a solution. If the mentioned condition is not met, the heuristic algorithm generates another potential solution.

## 4. Hybrid Algorithm

In order to solve the inverse problem of identifying objects in the considered region, it is necessary to find the minimum of the objective function (1). Due to the fact that the task is a non-continuous and non-differentiable optimization problem, we cannot use the classical gradient methods to find the minimum of the function. There is no analytical formula of objective function. Therefore, two non-gradient methods were used, combining them into one hybrid algorithm. These methods include, on the one hand, the meta-heuristic optimization algorithm, and the deterministic Hook-Jeeves (HJ) numerical method. This approach is new to this type of problem. The idea is to find an approximate initial solution using a meta-heuristic algorithm, which prevents falling into the so-called local minima, and then, using the HJ method, searching for a precise solution. Thanks to this approach, we use the best features of both algorithms to create a hybrid algorithm. The algorithms used in the paper are described in the next sections.

### 4.1. Group Teaching Optimization Algorithm (GTOA)

The proposed GTOA algorithm is inspired by the process of teaching a group of students. To better understand the mechanism of this method, first a brief explanation of the basics of GTOA is proposed. The main idea is to teach students according to their abilities. In other words, the teacher should formulate appropriate teaching methods for students based on their various abilities. More precisely, group teaching aims to emphasize the subjectivity of students, i.e., adapting school education to the dissimilarity of students by offering different courses and teaching methods. In fact, students differ from each other in such characteristics as: intelligence, attitude to learning, ability to learn, economic conditions and predispositions. Thus, although group teaching is an effective way to improve the overall quality of students, in practice there is no uniform mode of group teaching.

The idea of the proposed GTOA is to improve the knowledge of the all students in class by simulating the mechanism of group teaching. Given the differences between students, implementing group teaching in practice is quite complicated. In order to adapt the idea of group teaching to be used as an optimization technique, a simple model is built, which is based on the following principles:

- The only difference between students is the ability to absorb knowledge. The greater the differences in this ability among students, the greater the challenge for the teacher in formulating effective teaching methods.
- A good teacher tends to pay more attention to students with poor ability to learn than to students with strong ability to absorb knowledge.
- In free time, a single student can gain knowledge through independent study and interaction with other students.
- Good allocation of teachers to different groups of students is important in the process of improving the knowledge of the latter.

The proposed model of group teaching includes four main stages: teacher allocation stage, ability grouping stage, teacher stage, and student stage. Next, each of the stages is presented in detail, described with a mathematical formula.

#### 4.1.1. Ability Grouping Stage

In general, the level of knowledge of students in the classroom is normally distributed with the probability density function (PDF):

$$f_{\mu ,\sigma }\left(x\right)=\frac{1}{\sigma \sqrt{2\pi }}\exp\left(\frac{-{\left(x-\mu \right)}^{2}}{2\sigma^{2}}\right).$$

In the above formula, the symbol μ denotes the average level of knowledge in the class, and σ is the standard deviation reflecting the differences in the level of knowledge among students. A good teacher takes into account not only the average level of knowledge μ, but also the standard deviation σ. The goal is to increase the average knowledge μ as well as reduce the difference σ among the group. To achieve this goal, the teacher should develop an appropriate teaching plan for his students.

In order to better show the feature of group teaching, without losing generality, all students are divided into two small groups according to their ability to learn. Both groups are equally important in GTOA. Thus, both groups have the same or similar number of students. One group with a strong ability to absorb knowledge can be called an outstanding group. Another group with poor learning abilities can be called the average group. It is obvious that the standard deviation in both small groups is smaller than in large one. It is easier for the teacher to work in small groups where the level of knowledge of students is similar. In the case of GTOA, skill grouping is a dynamic process that is performed again after the learning cycle (after each iteration of the algorithm).

#### 4.1.2. Teacher Stage

In this stage, each student acquires knowledge from the teacher. The teacher creates different learning plans for the average group and the outstanding group in the proposed GTOA.

In this stage, for the outstanding group, due to their strong ability to absorb knowledge, the teacher focuses on improving the knowledge of the outstanding group as a whole, increasing its average. More specifically, the teacher can make an effort to improve the knowledge of all students in classroom. In this way, a student in an outstanding group can gain knowledge according to the following formula:

(2) $$x_{ts,j}^{t+1}=x_{j}^{t}+\alpha \left(T^{t}-w\left(\beta M^{t}+\left(1-\beta \right)x_{j}^{t}\right)\right).$$

In Formula (2), the following notations are adopted: $x_{ts,j}^{t+1}$ is the knowledge of the *j*-th student after completing the teaching stage, $x_{j}^{t}$ is the knowledge of the student before the teaching stage (in a given cycle), and $T^{t}$ is the knowledge of the teacher in iteration *t*. By $M^{t}$ we denote the average knowledge of students in the group in a given iteration and define by the formula:

(3) $$M^{t}=\sum_{j=1}^{N}x_{j}^{t},\;\;\;\;\;N-\mathrm{number}\;\mathrm{of}\;\mathrm{students}\;\mathrm{in}\;\mathrm{the}\;\mathrm{group}.$$

The meaning of the symbol $T^{t}$ is presented next, when discussing the teacher allocation stage. The coefficients α,β are random numbers in the range [0,1], and *w* is the teaching parameter (usually *w* is 1 or 2 [16]).

In the teacher stage, the teacher pays more attention to the average group than to the outstanding one, taking into account the poor ability of the average group to absorb knowledge. In the case of an outstanding group, students could also acquire knowledge from amongst themselves, while in the average group, the teacher plays a very important role. Hence, this process can be described mathematically as follows:

(4) $$x_{ts,j}^{t+1}=x_{j}^{t}+2d\left(T^{t}-x_{j}^{t}\right),$$

where d∈[0,1] is a random number.

If a given student after the teaching stage has not increased his knowledge, then in the GTOA algorithm, we do not update the solution. Obviously, when a solution has been corrected, we accept it.

#### 4.1.3. Student Stage

The student can also acquire knowledge without the support of the teacher. Two main ways are possible, by self-learning or by interacting with other students. We can express this process mathematically as follows:

(5) $$\begin{array}{cc} x_{ss,j}^{t+1} & =x_{ts,j}^{t+1}+\mathrm{sgn}\left(j,k\right)\;r_{1}\left(x_{ts,j}^{t+1}-x_{ts,k}^{t+1}\right)+r_{2}\left(x_{ts,j}^{t+1}-x_{j}^{t}\right),\\ \mathrm{sgn}(j,k) & =\left\{\begin{array}{cc} 1, & f\left(x_{ts,j}^{t+1}\right)<f\left(x_{ts,k}^{t+1}\right),\\ -1, & f\left(x_{ts,j}^{t+1}\right)\geq f\left(x_{ts,k}^{t+1}\right), \end{array}\right. \end{array}$$

where $x_{ss,j}^{t+1}$ is the knowledge of *j*-th student after the student stage, $x_{ts,j}^{t+1}$ is the knowledge of *j*-th student after the teacher stage, $x_{j}^{t}$ denotes the knowledge of *j*-th student before the learning process with a teacher, and $r_{1},r_{2}$ are random numbers in the range [0,1]. The index k≠j in Equation (5) is chosen randomly from the remaining indices. The second term in Formula (5) symbolizes learning from another student, while the meaning of the third term relates to self-learning. In the end of this stage, it is decided whether a student has acquired more knowledge in this stage of learning (the student moves on to the next cycle):

(6) $$x_{j}^{t+1}=\left\{\begin{array}{cc} x_{ts,j}^{t+1}, & f\left(x_{ts,j}^{t+1}\right)<f\left(x_{ss,j}^{t+1}\right),\\ x_{ss,j}^{t+1}, & f\left(x_{ts,j}^{t+1}\right)\geq f\left(x_{ss,j}^{t+1}\right). \end{array}\right.$$

#### 4.1.4. Teacher Allocation Stage

In the last stage, the teacher allocation mechanism is presented. It is important for improving the knowledge of students. The teacher $T^{t+1}$ in the iteration t+1 is chosen based on the formula:

(7) $$T^{t+1}=\left\{\begin{array}{c} x_{first}^{t},\;\;\;f\left(x_{first}^{t}\right)<f\left(\frac{x_{first}^{t}+x_{second}^{t}+x_{third}^{t}}{3}\right),\\ \frac{x_{first}^{t}+x_{second}^{t}+x_{third}^{t}}{3},\;\;\;\;\;\;f\left(x_{first}^{t}\right)\geq f\left(\frac{x_{first}^{t}+x_{second}^{t}+x_{third}^{t}}{3}\right), \end{array}\right.$$

where $x_{first}^{t},x_{second}^{t},x_{third}^{t}$ are the three best students in cycle *t*.

Information regarding possible applications of the GTOA can be found in [16,17,18]. The pseudocode of the GTOA algorithm is shown in Algorithm 1.
**Algorithm 1** Group Teaching Optimization Algorithm (GTOA) pseudocode1:**Initialization part**2: Parameter initialization.3: Generating the starting population randomly.4:**Iterative part**5: Evaluation of knowlegde for each student in the population and determining the best solution.6: Check the algorithm termination criterion (for example, the maximum number of iterations). If the number of current iteration is less than the maximum, go to the next step, otherwise end the algorithm and return the best solution.7: Teacher allocation stage. Determine best three students, as well as teacher according to the Equation (7).8: Ability grouping stage. Divide population into two groups (average and outstanding) according to the value of fitness function (knowledge level).9: Teacher stage. Transform the solutions in both groups according to the Equations (2) and (4).10: Student stage. Transform the solutions according to the Equations (5) and (6).11: Create a new population with the number t+1 and increase the iteration iterator. Go to step 5.

### 4.2. Hook-Jeeves Method

The Hook-Jeeves algorithm is a deterministic numerical optimization method for finding the minimum of the objective function. This algorithm belongs to the so-called derivative-free search, or black-box search groups of optimization methods. These methods are determined by a vector of decision variables: trial and working. Thus, we can distinguish two ways of moving in the considered area:

- The trial method, which is used to study the behavior of the function in a small selected region with the use of trial steps along all directions of the orthogonal basis.
- The working method, which consists of moving in a strictly determined way to the next area in which the next trial stage is considered. This happens only when at least one of the performed steps was successful (this is the step as a result of which the objective function decreased its value).

We define the step as follows:

(8) $$x=x_{0}+\tau b^{k},$$

where $x_{0}\in R^{n}$ is a start point, τ is the length of the step, and $b^{k}\in R^{n}$ is *k*-th vector of the basis.

If none of the performed steps were successful, then we return to the previously selected area, and the search cycle starts again with a reduced step length. In order to describe the HJ method, let us introduce the following notations:

*n*—number of searched parameters,
$\left[b^{1},b^{2},\ldots ,b^{n}\right]$—orthogonal base of vectors,
$\left[\tau^{1},\tau^{2},\ldots ,\tau^{n}\right]$—step lengths, which can be different for each variable,
β∈(0,1)—parameter narrowing the stride length,
ε—accuracy of calculations.

More information about Hook-Jeeves method can be found in [19,20,21,22]. Algorithms 2 and 3 illustrate subsequent steps of the algorithm.

**Algorithm 2** Hook-Jeeves (HJ) method–trial stage pseudocode
1: Calculate the value of the objective function for the starting point $x_{0}=x_{old}$, $v_{0}=f\left(x_{old}\right)$.2: Perform a test step along the *i*-th direction i(i=1,2,…,n):$$x_{new}=x_{old}+\tau b^{i},$$
and calculate the value of the objective function $v=f(x_{new})$.3: Check if the step is successful, (i.e., $v<v_{0}$). If yes, substitute *v* for $v_{0}$, then go to step 6. If no, then go to step 4.4: Perform a test step in the opposite direction:$$x_{new}=x_{old}-\tau b^{i},$$
and calculate the value of the objective function for new point $v=f(x_{new})$.5: Check if the step is successful. If yes, substitute *q* for $q_{0}$ and proceed to step 6, otherwise leave the current point unchanged, i.e., replace $x_{old}$ for $x_{new}$.6: Test whether steps have been taken in all directions of the orthogonal basis. If not, select another direction from the database and repeat the steps from step 2, otherwise go to step 7.7: Check if there are successful steps in the run of the search cycle. If yes, substitute $x_{B}$ for the point $x_{B_{0}}$ and $x_{new}$ for the base point $x_{B}$ and proceed to the working stage (see Algorithm 3). Otherwise, follow step 8.8: If the end-of-algorithm criterion is not met (step length τ less than accuracy ε), check whether the current iteration is the first iteration. If yes, change the starting point and repeat the steps from point 1. If not, return to the previously searched area and reduce the stride length, i.e., τ=β·τ and start the procedure from the beginning, i.e., from point 1.


**Figure 4:**
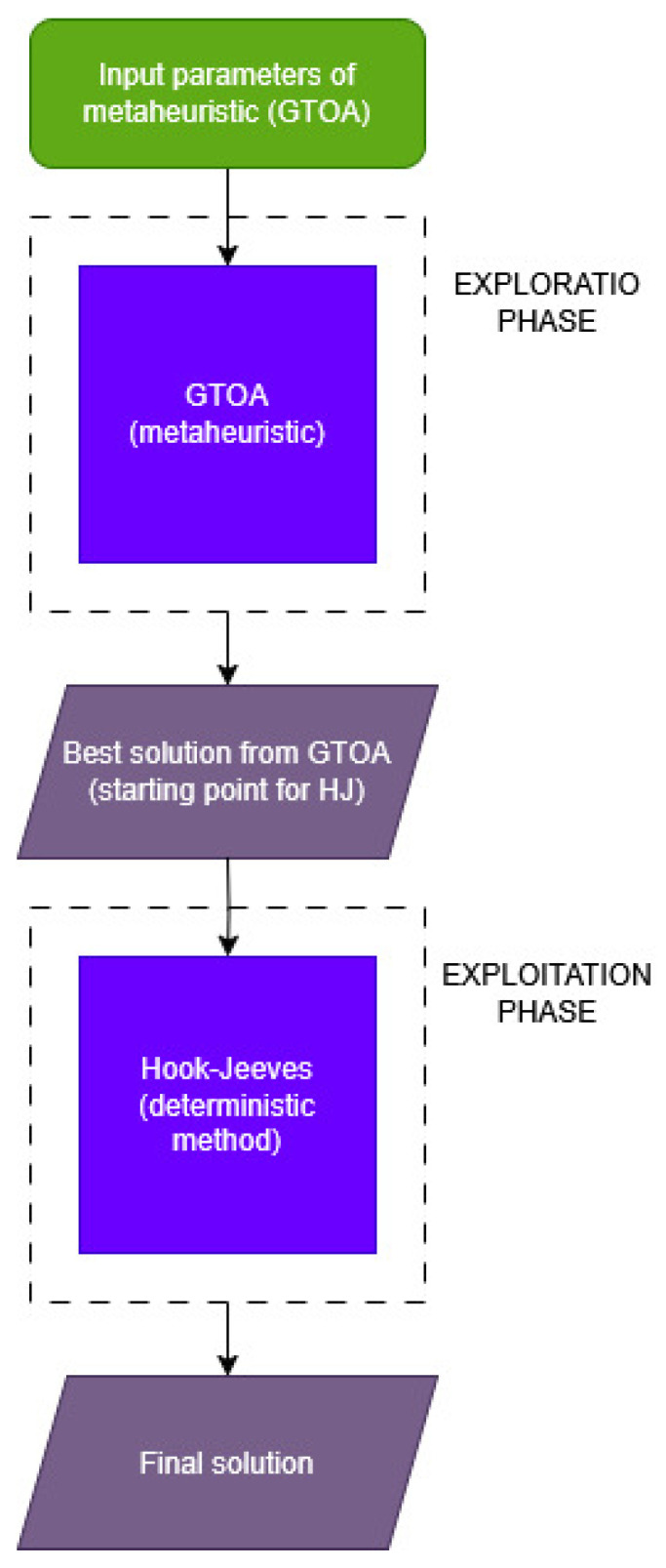
Block diagram of the hybrid (GTOA + HJ) algorithm.

Figure 4 shows the schematic operation of the hybrid algorithm. The solution obtained from GTOA serves as the starting point for the Hook-Jeeves algorithm.

**Algorithm 3** Hook-Jeeves (HJ) method–working stage pseudocode
1: Proceed with the working stage according to the formula:$$x_{new}=2*x_{B}-x_{B_{0}}$$2: Substitute $x_{B}$ for $x_{B_{0}}$ and return to the trial method.


## 5. Results

As mentioned earlier, in this paper, we employ a hybrid approach combining two types of algorithms. This is discussed in Section 4.1 where the heuristic algorithms is presented, and in Section 4.2 where a deterministic algorithm is introduced. The aim of this approach is to eliminate the drawbacks of both groups of algorithms. Heuristic algorithms quickly identify the area where the sought-after solution is located, but take a relatively long time to find a solution in this area that meets the desired accuracy. Deterministic algorithms, on the other hand, have difficulty identifying the area containing the solution, but if this area is narrowed down for them, they quickly obtain a solution with the desired accuracy. Therefore, for the given problem, we initially run a heuristic algorithm, which quickly provides an approximate solution close enough to the exact solution. Then, the deterministic algorithm is executed, with the initial solution being the result obtained from the heuristic algorithm.

To assess the usefulness and stability of the discussed method, we conducted numerous simulations. To illustrate the operation of the proposed method, below, we present two examples illustrating the successive stages of determining the reconstruction of the interior of the examined object.

### 5.1. Example 1

Let us assume that the examined object (or more precisely, one of its cross-sections) is described by the function:

$$f\left(x,y\right)=\left\{\begin{array}{cc} 1, & \left(x,y\right)\in \left([-0.6,-0.4]\times [-0.2,0.6]\cup [-0.4,-0.2]\times [-0.6,0]\right),\\ 3, & (x,y)\in [0.2,0.6]\times [0.4,0.6],\\ 0, & \mathrm{in}\;\mathrm{other}\;\mathrm{points}\;\mathrm{of}\;\mathrm{the}\;\mathrm{domain}, \end{array}\right.$$

where the domain is the set ${\left[-1,1\right]}^{2}$. This means that the exact solution to this problem is a vector from the space $R^{15}$:

$$\left[-0.6,-0.2,-0.4,0.6,1,-0.4,-0.6,-0.2,0,1,0.2,0.4,0.6,0.6,3\right].$$

**Figure 5:**
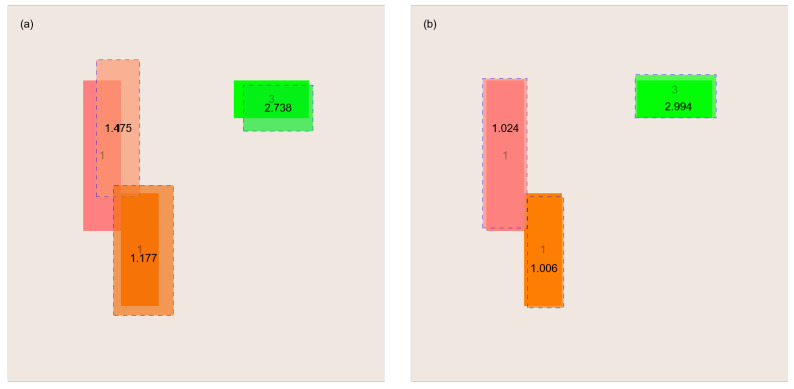
A sample process of reconstructing the interior of the examined object—(**a**) solution obtained using the GTOA algorithm and (**b**) solution obtained using the HJ algorithm, for which the solution from figure (**a**) served as the initial approximation.

By running the heuristic GTOA algorithm, we quickly obtain an approximate solution, which serves as the initial approximation for the deterministic HJ algorithm. In turn, with a good initial approximation, the HJ algorithm quickly finds satisfactory final approximate solutions. A graphical illustration of these searches is presented in Figure 5.

### 5.2. Example 2

Let assume that the examined object is described by the function:

$$f\left(x,y\right)=\left\{\begin{array}{cc} 1, & (x,y)\in [-0.9,-0.5]\times [0,0.4],\\ 2, & \left(x,y\right)\in \left([-0.4,0.3]\times [-0.5,-0.2]\cup [0.1,0.7]\times [-0.7,-0.5]\right),\\ 3, & (x,y)\in [0.2,0.5]\times [0.2,0.9],\\ 0, & \mathrm{in}\;\mathrm{other}\;\mathrm{points}\;\mathrm{of}\;\mathrm{the}\;\mathrm{domain}, \end{array}\right.$$

where the domain is the set ${\left[-1,1\right]}^{2}$. This means that the exact solution to this problem is a vector from the space $R^{20}$:

$$\left[-0.4,-0.5,0.3,-0.2,2,0.1,-0.7,0.7,-0.5,2,-0.9,0,-0.5,0.4,2,0.2,0.2,0.5,0.9,3\right].$$

It should be noted that, in general, the solution in the form of the vector presented above is ambiguous. Theoretically, it is possible to indicate an unlimited number of different vectors that would generate an exact solution. This is because the order of the rectangles is irrelevant, so we have n! different vectors for the case of reconstructing *n* rectangles. This ambiguity can be easily eliminated by treating the solution not as a vector, but as a set of vectors of length 5 (such sets can be easily sorted, thanks to which previously different solutions will now become equivalent solutions).

**Figure 6:**
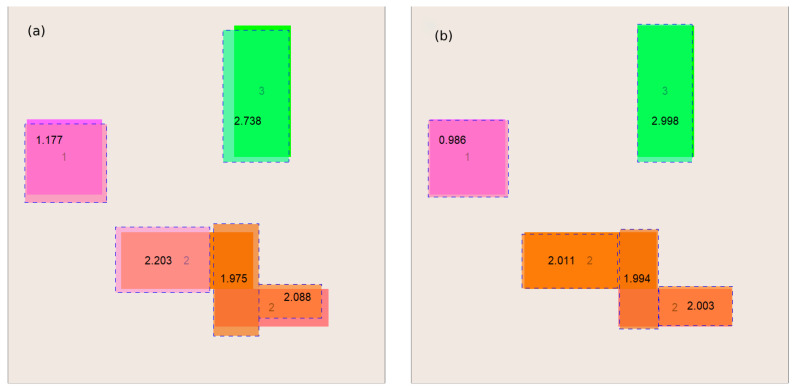
A sample process of reconstructing the interior of the examined object—(**a**) solution obtained using the GTOA algorithm and (**b**) solution obtained using the HJ algorithm, for which the solution from figure (**a**) served as the initial approximation.

Another cause for ambiguity is the number of rectangles being recreated. The area consisting of one rectangle can equally well be represented as any number of rectangles—e.g., pixels making up the input rectangle. We solved this problem in the following way: the procedure for reconstructing the interior of the examined object is initially started for the smallest possible number of rectangles, and then, if a solution cannot be found, we increase the number of rectangles sought by one until a solution is found. This situation is illustrated by example 2, where we were forced to look for a solution consisting of five rectangles, although four is sufficient. A graphic illustration of the discussed case can be found in Figure 6.

### 5.3. Example 3

As a third example, we present a case representing a wall with a non-uniform structure containing voids inside. The fragment of the wall is a rectangle with two vertices at the points (−1.5,−3), (1.5,−3), (1.5,3) and (−1.5,3). The voids inside this area are four polygons defined as a sequence of consecutive vertices:

$$\begin{array}{cc} p_{1}= & \left((-0.3,-2),(-0.3,-1.9),(0.5,-1.5),(0.5,-1.8)\right),\\ p_{2}= & \left((-0.5,1),(-0.4,0.1),(0.3,0.2),(0.5,0.7)\right),\\ p_{3}= & \left((-0.5,2.1),(-0.5,1.6),(0.3,1.7),(0.5,2.2)\right),\\ p_{4}= & \left((-0.4,-0.5),(-0.5,-0.64),(0.35,-0.8),(0.4,-0.3)\right). \end{array}$$

**Figure 7:**
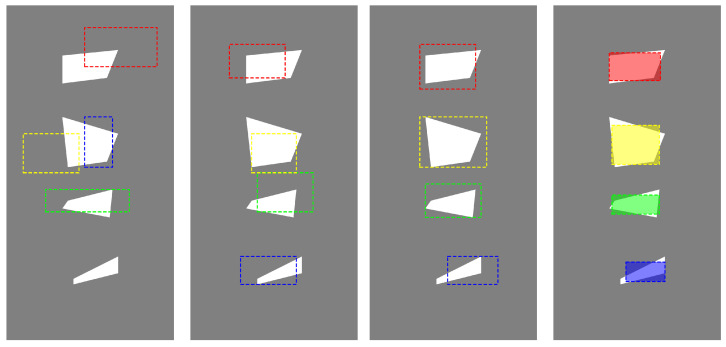
An example of the process of reconstructing the interior of a non-uniform wall, the subsequent figures, starting from the left, show the results after 10, 20, and 50 iterations of the GTOA algorithm and after applying the H-J algorithm.

In the first approach, the solution was sought in the form of four rectangles. The partial and final results are presented in Figure 7, where the subsequent figures show the considered area and the solution sought, marked with a dashed line, after 10, 20 and 50 iterations of the GTOA algorithm, respectively. The result after 50 iterations is the starting point for the deterministic H-J algorithm. The final result is presented in the form of shaded rectangles. The benefit of using a deterministic algorithm is that we obtain a solution to the problem much faster, because heuristic algorithms usually lead to a solution close to the exact one relatively quickly, but quite large iterations must be performed to obtain satisfactory solutions that converge to the exact solution. A deterministic algorithm with an appropriate starting point converges much faster.

**Figure 8:**
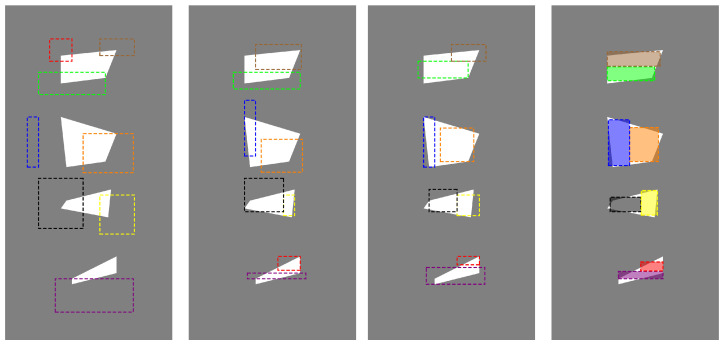
An example of the process of reconstructing the interior of a non-uniform wall, the subsequent drawings, starting from the left, show the results after 10, 100, and 250 iterations of the GTOA algorithm and after applying the H-J algorithm.

As a second case, we present the same example in which we are looking for eight rectangles as a solution to the problem. In this case, the results are presented in Figure 8. The following figures, starting from the left, show approximate solutions after 10, 100, and 250 iterations, respectively. In this case, with a larger number of parameters, the process converges much slower and the heuristic algorithm, in acceptable computation time, only leads to a solution that is a good starting point for the deterministic method. The H-J algorithm leads to a satisfactory solution much faster than the heuristic algorithm. The result is shown similarly to the previous case as shaded rectangles.

In Table 1, the quality of the obtained results and their computation times are compared for the heuristic GTOA algorithm and the hybrid GTOA+H-J algorithm. The error presented in the Table is understood as the percentage of the misclassified area relative to the total area, and related time expresses the ratio between the current example and the longest computation time included in the Table, which was obtained for the GTOA algorithm with n=8. The results presented in each of the compared cases show a clear acceleration of the hybrid algorithm compared to the heuristic algorithm that is part of the hybrid algorithm. This is because heuristic algorithms tend to approach the solution relatively quickly in the initial iterations and then require significantly more iterations to achieve a satisfactory solution. This means that the use of a deterministic algorithm allows for obtaining a satisfactory solution much faster. Naturally, in the presented example, as the number of sought rectangles *n* increases, the error of the obtained solution decreases, but due to the geometry of the presented area, it will never reach zero with a finite number of applied rectangles. It will only converge to it as *n* increases.

### 5.4. Comparison of Reconstruction Methods

The proposed method was compared with other techniques appropriate for this type of problem. The comparison is presented in Table 2, which includes the operational principles of each method, their level of invasiveness, and accuracy. The proposed approach demonstrates good performance relative to the other methods. The hammer tapping technique was excluded from the table due to its subjectivity and the low quality of the results it produces. This method is not recommended for historical masonry structures. A key advantage of the method presented in this study is its ability to detect all voids in a single examination, regardless of their number or location—an aspect that poses a limitation for methods such as Ground Penetrating Radar (GPR) and Ultrasonic Testing.

Additionally, Table 3 presents a comparison of the key features of tomography in the context of analytical and algebraic tomography with an incomplete data set. The comparison clearly indicates that only algebraic tomography can be adapted for investigating wall structures, primarily due to the limited range of available projection angles. The mathematical model underlying the proposed method is based on the algebraic tomography framework; however, to accelerate computations, it has been reformulated as an optimization problem. The optimization process was carried out using heuristic techniques. Combining heuristics with a deterministic method (a hybrid approach) enabled a significant reduction in computation time.

## 6. Conclusions

Research on historical buildings is often necessary for cognitive, renovation, or durability purposes. Often, the oldest (usually the lowest) parts of such walls cannot be examined using invasive methods due to the risk of structural damage or the violation of conservation principles. Computed tomography (CT) offers a promising non-invasive alternative. However, due to size constraints and difficult accessibility, classical CT techniques based on complete data sets are either inefficient or unusable.

In this article, we proposed a non-classical hybrid approach to computed tomography using incomplete datasets. The combination of a heuristic algorithm, which quickly finds an initial approximation, with a deterministic refinement algorithm allows for effective and efficient reconstruction. This approach avoids long computations of heuristic “fine-tuning” and addresses the main limitation of deterministic methods—the need for a good starting point.

The results showed that our hybrid method is significantly more efficient and stable than traditional ART-family algorithms, while maintaining accuracy. Importantly, this makes it suitable for real-world applications, such as diagnosing moisture levels in heritage buildings.

To fully realize this potential, future work should involve testing the method on actual tomographic images of moist masonry or other hydrated materials. As noted in recent studies on moisture propagation in porous, fractal-like structures such as rocks [1], modeling moisture behavior in complex internal geometries remains a challenge. Our method can potentially offer predictive power in such scenarios, especially where conventional models fail.

In conclusion, the presented approach can be a useful tool in heritage conservation and civil engineering, supporting early diagnosis of moisture-related degradation processes in hard-to-reach areas of historical structures.

## Figures and Tables

**Table 1:** Comparison of results and computation time for the application of the heuristic GTOA algorithm and the deterministic H-J method, where *n* denotes the number of sought rectangles, error expresses the percentage of the misclassified area relative to the total area, and related time expresses the ratio between the current example and the longest computation time included in the table (for the GTOA algorithm with *n* = 8).

Method	*n*	Iter		Error [%]	Related Time [%]
GTOA	4	120		2.43	13.93
	5	189		2.05	22.06
	6	345		1.78	37.51
	7	591		1.15	66.74
	8	890		0.75	100
GTOA+H-J	4	50	45	2.44	5.71
	5	63	52	2.04	7.11
	6	121	61	1.77	13.99
	7	194	82	1.14	22.25
	8	250	113	0.69	31.02

**Table 2:** Comparison of selected non-destructive methods for void detection in historic masonry.

Method	Principle	Invasiveness	Accuracy	Application	Ref.
Infrared Thermography (IRT)	Captures infrared radiation emitted from the wall surface.	Non-invasive	Medium	Detects voids, moisture, and thermal bridges.	[23]
Ground Penetrating Radar (GPR)	Emits EM waves and analyzes reflections from subsurface features.	Non-invasive	High	Locates voids, delaminations, and buried elements.	[24]
Ultrasonic Testing	Measures velocity or attenuation of sound waves through masonry.	Slightly invasive	Medium	Detects cracks, voids, and material inhomogeneities.	[25]
Approach to application in the article	Imaging of internal structures using multiple sources of penetrating radiation.	Non-invasive	High	Structural analysis of columns or masonry elements.	[26]
Electrical Resistivity Method	Measures electrical resistance, affected by moisture and material continuity.	Semi-invasive	Low–Medium	Preliminary moisture or void detection.	[27]

**Table 3:** Comparison between analytical and algebraic tomography (with incomplete data).

Criterion	Analytical Tomography	Algebraic Tomography (Incomplete Data)
Principle	Radon transform, filtered backprojection	Linear equations, iterative algorithms (e.g., ART, SIRT)
Required Number of Projections	High (full rotation needed)	Low (can work with limited-angle or sparse data)
Noise Resistance	Low	High (regularization possible)
Reconstruction Accuracy (Full Data)	High	High
Accuracy with Incomplete Data	Poor	Better (adaptive to missing data)
Computation Time	Short	Long (many iterations)
Implementation Complexity	Relatively simple (classic math)	Complex (numerical optimization)
Applications	Medical CT, industrial imaging with full access	Cultural heritage, archaeology, limited-access diagnostics
Limited-Angle Reconstruction	Very limited	Good

## Data Availability

Data are contained within the article.
